# The M3 Muscarinic Acetylcholine Receptor Promotes Epidermal Differentiation

**DOI:** 10.1016/j.jid.2022.06.013

**Published:** 2022-07-21

**Authors:** Junyan Duan, Charles Grando, Shuman Liu, Alex Chernyavsky, Jefferson K. Chen, Bogi Andersen, Sergei A. Grando

**Affiliations:** 1 Center for Complex Biological Systems, University of California, Irvine, Irvine, California, USA; 2 NSF-Simons Center for Multiscale Cell Fate Research, University of California, Irvine, Irvine, California, USA; 3 Department of Dermatology, School of Medicine, University of California, Irvine, Irvine, California, USA; 4 Division of Endocrinology, Department of Medicine, School of Medicine, University of California, Irvine, Irvine, California, USA; 5 Department of Biological Chemistry, School of Medicine, University of California, Irvine, Irvine, California, USA; 6 Institute for Immunology, University of California, Irvine, Irvine, California, USA

## Abstract

The M3 muscarinic acetylcholine receptor is predominantly expressed in the basal epidermal layer where it mediates the effects of the autocrine/paracrine cytotransmitter acetylcholine. Patients with the autoimmune blistering disease pemphigus develop autoantibodies to M3 muscarinic acetylcholine receptor and show alterations in keratinocyte adhesion, proliferation, and differentiation, suggesting that M3 muscarinic acetylcholine receptor controls these cellular functions. *Chmr3*^*−/−*^ mice display altered epidermal morphology resembling that seen in patients with pemphigus vulgaris. In this study, we characterized the cellular and molecular mechanisms through which M3 muscarinic acetylcholine receptor controls epidermal structure and function. We used single-cell RNA sequencing to evaluate keratinocyte heterogeneity and identify differentially expressed genes in specific subpopulations of epidermal cells in *Chmr3*^*−/−*^ neonatal mice. We found that *Chmr3*^*−/−*^ mice feature abnormal epidermal morphology characterized by accumulation of nucleated basal cells, shrinkage of basal keratinocytes, and enlargement of intercellular spaces. These morphologic changes were associated with upregulation of cell proliferation genes and downregulation of genes contributing to epidermal differentiation, extracellular matrix formation, intercellular adhesion, and cell arrangement. These findings provide, to our knowledge, previously unreported insights into how acetylcholine controls epidermal differentiation and lay a groundwork for future translational studies evaluating the therapeutic potential of cholinergic drugs in dermatology.

## INTRODUCTION

The muscarinic acetylcholine (ACh) receptors are single-subunit transmembrane glycoproteins of five subtypes (M1–M5). On ACh binding, muscarinic ACh receptors (mAChRs) stimulate interactions of G proteins with signal transducing enzymes, leading to changes in second messengers, ion concentrations, and the modulations of protein kinase activities. Within the epidermis, ACh is released in an autocrine and paracrine manner, and the different mAChR subtypes are combinatorically expressed in a differentiation-specific manner, suggesting complex epidermal regulation (reviewed in Grando [2012, 2006]).

Previous research, primarily using in vitro models, has implicated mAChRs in multiple keratinocyte (KC) functions, including cell–cell and cell–substrate adhesion, migration, proliferation, and differentiation (reviewed in Grando [2012, 2006]). Successful use of topical muscarinic agonist pilocarpine to heal skin erosions in patients with the autoimmune blistering disease pemphigus and the disappearance of psoriatic lesions due to systemic therapy with the muscarinic antagonist atropine highlight the importance of understanding the in vivo effects of the mAChR pathway (reviewed in Grando [2012]).

Blocking mAChRs in undifferentiated human KC monolayers significantly increased cell numbers and inhibited differentiation when KCs were exposed to differentiation-inducing agents (reviewed in Grando [2012]). Blocking of mAChRs in organotypic cultures of human epidermis also altered epidermal differentiation as evidenced by increased expression of injury markers keratin (K)6 gene *K6/K16* and decreased expression of the differentiation markers *K10* and *FLG* as well as cell adhesion proteins (Kurzen et al., 2006). The outcome was cell–cell separation (acantholysis) in the basal and lower suprabasal layers with defective epidermal barrier and cell death through intrinsic activation of apoptosis (Kurzen et al., 2006). Acantholysis caused by blockade of KC mAChRs was associated with increased phosphorylation of the adhesion molecules E-cadherin, desmoglein 3, and β- and γ-catenins, suggesting that regulation of KC cell–cell adhesion through the mAChR class predominantly involves changes in the phosphorylation of intercellular adhesion molecules (Nguyen et al., 2004), but the effects of specific mAChRs subtypes on in vivo epidermal differentiation are incompletely understood.

Previous studies of the role of cholinergic autocrine and paracrine regulation of non-neuronal cells through mAChRs have given contradictory results about the role of specific mAChR subtypes in cell proliferation, suggesting that mAChRs induce either cell cycle arrest (Chang, 2001; Jeng et al., 1999; Kurzen et al., 2006; Thangjam and Kondaiah, 2009) or promote cell proliferation (Arredondo et al., 2003). Most of these experiments were performed with cultured human KCs rather than in an animal model and employed muscarinic ligands that lack selectively to specific mAChR subtypes. Therefore, the precise effects of specific mAChR subtypes on in vivo epidermal cell proliferation and molecular mechanisms mediating the physiologic regulation by ACh remain to be identified.

To identify the cell types controlled by non-neuronal ACh in the murine epidermis, discern major cell functions, and identify the principal regulatory mechanisms in this study, we employed single-cell RNA-sequencing (scRNA-seq) technology and the receptor-knockout (KO) mice. We focused on the M3 mAChR subtype (*Chrm3*), the major mAChR mediating ACh signals in basal KCs. CHRM3 is preferentially coupled to the activation of pertussis toxin–insensitive G proteins of the G α q/11 family, which activate phospholipase C and produce inositol 1,4,5-triphosphate and diacylglycerol. These second messengers elicit the activation of protein kinase C and trigger the release of calcium ion from intracellular stores. CHRM3 is one of the major antigens targeted by autoantibodies in severe pemphigus (Chernyavsky et al., 2020, 2019; Kalantari-Dehaghi et al., 2013; Lakshmi et al., 2017; Sinha, 2011). Patients with pemphigus develop intra-epidermal cell–cell detachment (acantholysis) above the basal cell layer, blisters, and nonhealing erosions (reviewed in Grando [2006]).

We found that *Chrm3*^*−/−*^ neonatal mice feature abnormal epidermal morphology characterized by an increased number of basal cells and epidermal thickness. Intercellular spaces in the basal cell layer are increased, consistent with decreased cell–cell adhesion. These morphologic changes were associated with upregulation of cell proliferation genes and downregulation of epidermal differentiation genes as well as downregulation of the expression of genes contributing to extracellular matrix formation, intercellular adhesion, and cell arrangement. These findings provide new insights into the molecular mechanisms by which ACh regulates epidermal development and lay the groundwork for translational studies on cholinergic drugs in dermatology.

## RESULTS

### Altered intercellular cohesion of basal cells and epidermal hyperplasia in neonatal *Chrm3*^*−/−*^ mice

**Figure 1.**
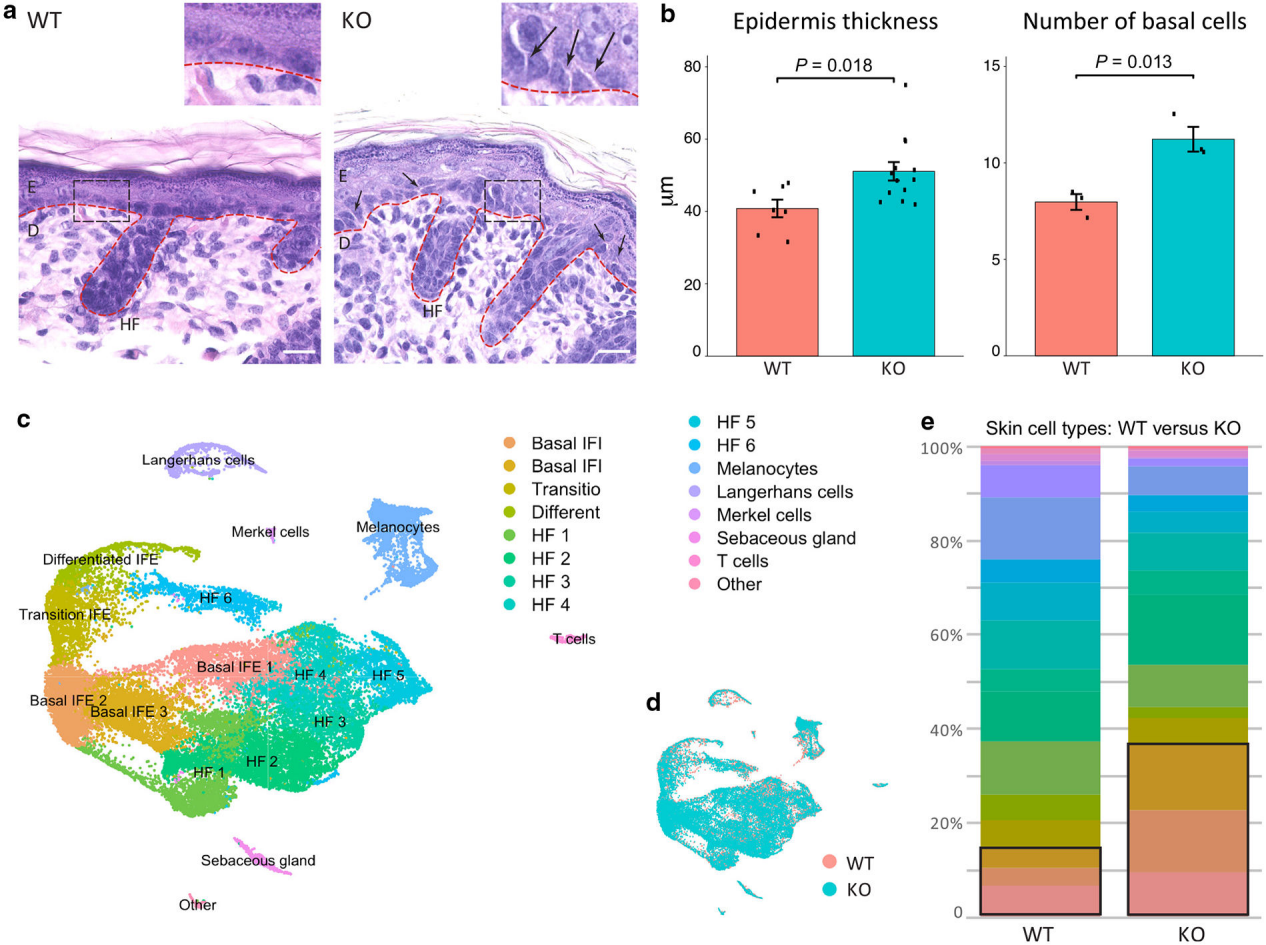
*Chrm3*^*−/−*^ thickens mouse epidermis. (**a**) The differences between epidermal morphology between WT versus Chrm3^(−/−)^ mice were manifested by an increased thickness of the basal layer comprised of crowded and slightly shrunk keratinocytes in KO mice. Bar = 10μm. (**b**) Quantitative analysis of the differences in epidermal morphology between WT and Chrm3^(−/−)^ mice showed an increase in both the thickness of epidermis and the number of nucleated basal cells within IFE in KO mice. *P* - value was calculated from two-tailed t-test. (**c**) UMAP showing the clusters of seven major epidermal cell types, including IFE, HF, melanocytes, Langerhans cells, Merkel cells, sebaceous gland, and T cells. The marker gene list is shown in Supplementary Table S1. (**d**) All the seven epidermal cell types are present in both WT and Chrm3^(−/−)^ epidermis. (**e**) WT and Chrm3^(−/−)^ Cellular composition of epidermis. The black box highlights the basal populations, which are increased in the Chrm3^(−/−)^ epidermis. HF, hair follicle; IFE, interfollicular epidermis; KO, knockout; UMAP, Uniform Manifold Approximation and Projection; WT, wild-type.

We studied postnatal day (P) 0 wild-type (WT) and *Chrm3*^*−/−*^ mice to define the epidermal role of ACh acting through CHRM3 (Figure 1a). In the epidermis of WT mice, basal layer KCs form a single row of polygonal epithelial cells with indistinct cell borders; the intercellular spaces are invisible. By contrast, the epidermis of *Chrm3*^*−/−*^ mice contains an increased number of basal cells, giving the impression of crowding of basal KCs (n = 3–3, *P* = 0.013) (Figure 1b). The overall epidermal thickness is also increased in *Chrm3*^*−/−*^ mice (n = 7–13, *P* = 0.018) (Figure 1b). Basal layer cells in *Chrm3*^*−/−*^ mice also appear smaller with distinct cell borders, revealing intercellular spaces (Figure 1a, arrows). At a higher magnification, cellular bridges crossing the intercellular spaces can be seen in the *Chrm3*^*−/−*^ epidermis (Figure 1a). Taken together, these results suggest that lack of *Chrm3* signaling is associated with decreased cell–cell adhesion in the basal layer and increased cell proliferation leading to epidermal hyperplasia.

### Expansion of stem and progenitor cells in the *Chrm3*^*−/−*^ mouse neonatal epidermis

To understand the cellular and transcriptomic changes underlying the epidermal abnormalities described earlier, we collected dorsal skin from *Chrm3*^*−/−*^ P0 C57BL/6J mice (n = 2), isolated epidermal cells, and performed scRNA-seq. In total, 19,486 cells passed the quality control and were included in the data analysis. To allow for comparison with normal neonatal epidermis, we integrated the *Chrm3*^*−/−*^ dataset with our previously published scRNA-seq data of 13,353 epidermal cells similarly collected from WT P0 C57BL/6J mouse epidermis (n = 2) (Lin et al., 2020). The integrated dataset contains 32,839 cells (Figure 1c).

Seven major epidermal cell types were identified in the dataset: interfollicular epidermis (IFE) KCs, hair follicle KCs, sebaceous gland cells, Merkel cells, melanocytes, Langerhans cells, and T cells (Figure 1c). The IFE readily forms five subpopulations. Using canonical marker genes (Joost et al., 2020, 2016; Lin et al., 2020), we identified three of them as basal cell clusters (basal IFE 1, n = 2,971; basal IFE 2, n = 3,041; basal IFE 3, n = 3,319), identified one as a transitional cell cluster (transition IFE, n = 1,895), and identified one differentiated cell cluster (differentiated IFE, n = 1,188). These IFE cluster assignments correspond well to our previous study on the role of GRHL3 in epidermal differentiation, showing several basal subtypes and a prominent cluster of cells transitioning between the basal and suprabasal layers (Lin et al., 2020).

*Chrm3* KO does not result in the loss of epidermal cell types or the formation of new cell types. The *Chrm3*^*−/−*^ cells do not form a unique cluster, and their positions in the Uniform Manifold Approximation and Projection (UMAP) generally overlap with those of the WT cells (Figure 1d). However, the cellular composition of the *Chrm3*^*−/−*^ epidermis is different from that of the WT. The proportion of IFE KCs in the skin and the total proportion of the basal IFE KC subpopulations almost doubled and tripled, respectively, in the *Chrm3*^*−/−*^ mice (Figure 1e). These data indicate that the increased epidermal thickness and cellularity of the *Chrm3*^*−/−*^ (Figure 1a) is due to the expansion of IFE stem and progenitor cells.

We also noted significant decreases in the proportion of Merkel cells (WT 1 = 0.14%, WT 2 = 0.19%, KO 1 = 0.06%, KO 2 = 0.06%) and Langerhans cells (WT 1 = 1.28%, WT 2 = 1.50%, KO 1 = 0.45%, KO 2 = 0.60%) in the *Chrm3*^*−/−*^ epidermis (Figure 1e), indicating that *Chrm3* may play a role in sensory and epidermal immune development.

### Expansion of stem cells, including cycling cells, and contraction of differentiated cells in the *Chrm3*^*−/−*^ IFE

**Figure 2.**
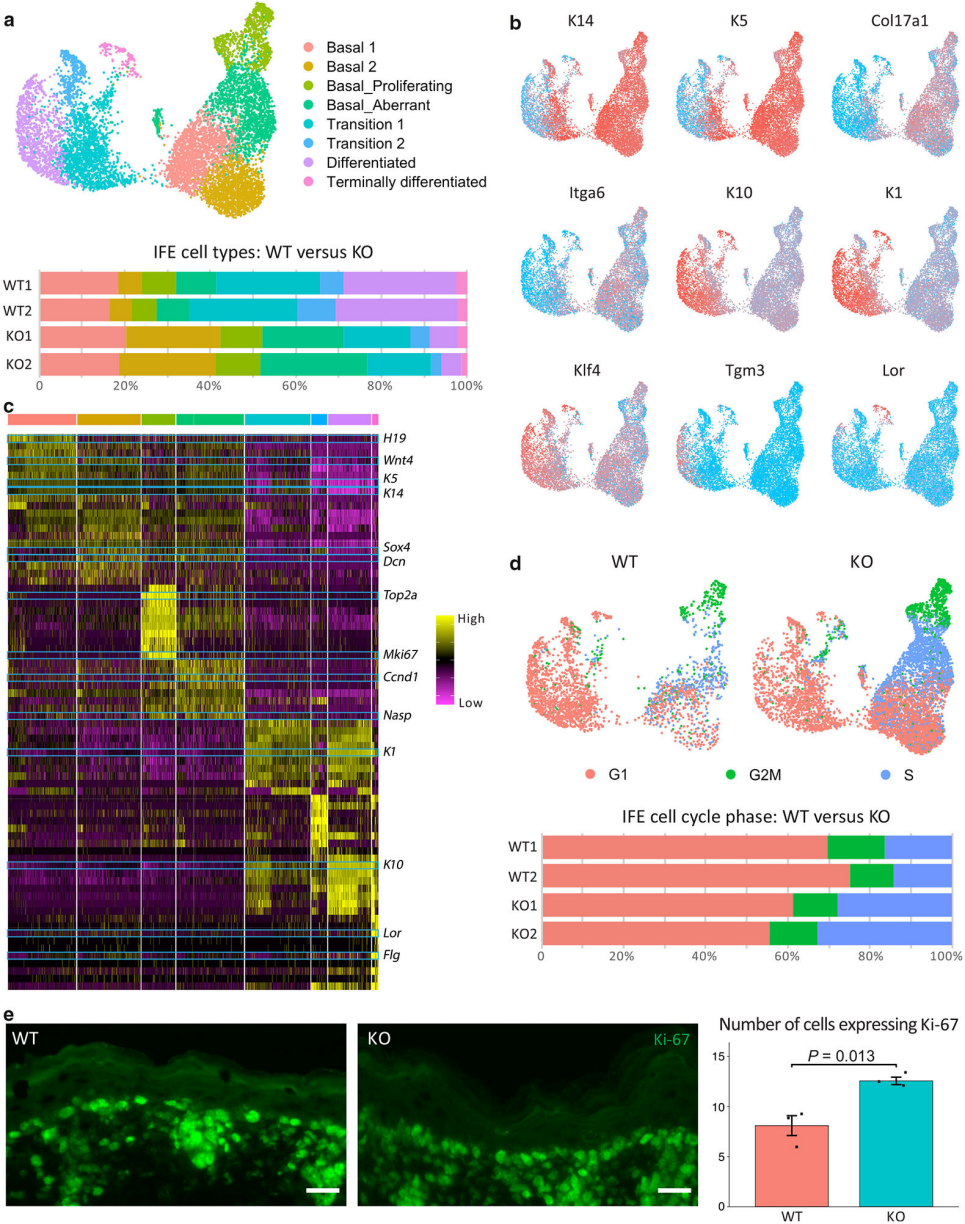
*Chrm3*^*−/−*^ expands the population of basal cells and cycling cells. (**a**) Upper: UMAP showing the eight clusters of the IFE, including four basal populations, two transition populations, one differentiated population, and a terminally differentiated population. Lower: Bar graph presenting the cellular composition of the WT and *Chrm3*^*−/−*^ IFE. (**b**) Expression of marker genes projected onto the UMAP to identify the basal cells (*K14, K5, Col17a1, Itga6*), differentiated cells (*K10, K1, Klf4, Tgm3*), and terminally differentiated cells (*Lor*). Red denotes high expression, and blue denotes low expression. (**c**) Heatmap showing the top 10 marker genes for each of the clusters defined in a. Blue boxes highlight some of the canonical marker genes. The complete marker gene list is shown in Supplementary Table S2. (**d**) Upper: cell cycle assignment projected onto the UMAP. Lower: bar graph showing the percentage of each cell cycle stage in the WT and *Chrm3*^*−/−*^ IFE. (**e**) A representative image of the epidermis WT and *Chrm3*^*−/−*^ mice staining with anti–Ki-67 antibody (bar = 20 μ m) as well as direct count of Ki-67–postive cells showing more positive cells in the basal layer of KO mice. *P*-value was calculated from two-tailed *t*-test. IFE, interfollicular epidermis; K, keratin; KO, knockout; Lor, loricrin; UMAP, Uniform Manifold Approximation and Projection; WT, wild-type.

To further examine the cellular abnormality in the neonatal *Chrm3*^*−/−*^ IFE, we computationally isolated the IFE KCs and performed subcluster analysis. Eight IFE clusters formed: four basal populations (basal 1 = 1,641, basal 2 = 1,524, basal proliferating = 813, and basal aberrant = 1,533), two transitional populations (transition 1: n = 1,574 and transition 2: n = 381), one differentiated population (differentiated = 1,034), and one terminally differentiated population (terminally differentiated = 249) (Figure 2a). The expression pattern of KC differentiation marker genes is consistent with that in previous studies establishing canonical markers for these populations (Joost et al., 2016; Lin et al., 2020) (Figure 2b). *K14, K5, Col17a1*, and *Itga6* are expressed at high levels in the basal populations, with the proliferating basal cells expressing additional proliferation marker genes such as *Mki67* and *Top2*. *K10, K1, Klf4*, and *Tgm3* are expressed in the differentiated populations. Loricrin gene *Lor* specifically marks the terminally differentiated population. The transition populations express marker genes for both basal and differentiated KCs but at lower levels (Figure 2b and c).

The increase in the proportion of basal cells in the *Chrm3*^*−/−*^ IFE is further confirmed in the subclustering analysis. All the four basal subpopulations are increased in the *Chrm3*^*−/−*^ epidermis, with the most significant increase in the basal 2 (WT 1 = 5.6%, WT 2 = 5.2%, KO 1 = 21.9%, and KO 2 =22.6%) and the basal aberrant clusters (WT 1 = 9.3%, WT 2 = 7.5%, KO 1 = 19.1%, and KO 2 =10.5%) (Figure 2a). Consistent with the pattern found in WT (Lin et al., 2020), *Chrm3*^*−/−*^ basal 1 expresses high levels of *H19, Igf2*, and *Wnt4*, whereas *Chrm3*^*−/−*^ basal 2 cluster expresses the marker genes of basal 1 but higher levels of decorin gene *Dcn*, a gene that encodes the extracellular matrix protein decorin, and *Sox4* (Figure 2c). Similar to the aberrant basal population found in the *Grhl3*^*−/−*^ epidermis (Lin et al., 2020), the basal aberrant cluster in *Chrm3*^*−/−*^ mice expresses the marker genes of both basal 1 and basal 2 but also some cell cycle marker genes such as *Ccnd1* and *Nasp*.

Cell cycle scoring using Seurat suggests that the basal aberrant cluster consists of cells in the S-phase. In fact, the proportion of cells expressing S-phase markers is twice higher in the *Chrm3*^*−/−*^ than in the WT IFE (WT 1 = 16.4%, WT 2 = 14.3%, KO 1 = 27.7%, KO 2 = 32.7%) (Figure 2d). Consistent with scRNA-seq findings, immunofluorescent staining of the epidermis from *Chrm3*^*−/−*^ and WT mice with anti–Ki-67 antibody revealed more positive cells (n = 3–3, *P* = 0.013) in the basal layer of KO mice than in the WT mice (Figure 2e).

In addition, the population of differentiated KCs is decreased in the *Chrm3*^*−/−*^ epidermis (WT 1 = 26.3%, WT 2 = 28.5%, KO 1 = 6.7%, KO 2 = 4.7%), whereas the terminally differentiated population is unchanged (WT 1 = 2.5%, WT2 = 2.1%, KO 1 = 2.0%, KO 2 = 2.3%) (Figure 2a). In summary, these data show the expansion of stem cells and a decrease in differentiated cells in the *Chrm3*^*−/−*^ IFE, indicating that *Chrm3* promotes differentiation of IFE stem cells.

### Downregulation of epidermal differentiation genes and upregulation of cell proliferation genes in the *Chrm3*^*−/−*^ epidermis

**Figure 3.**
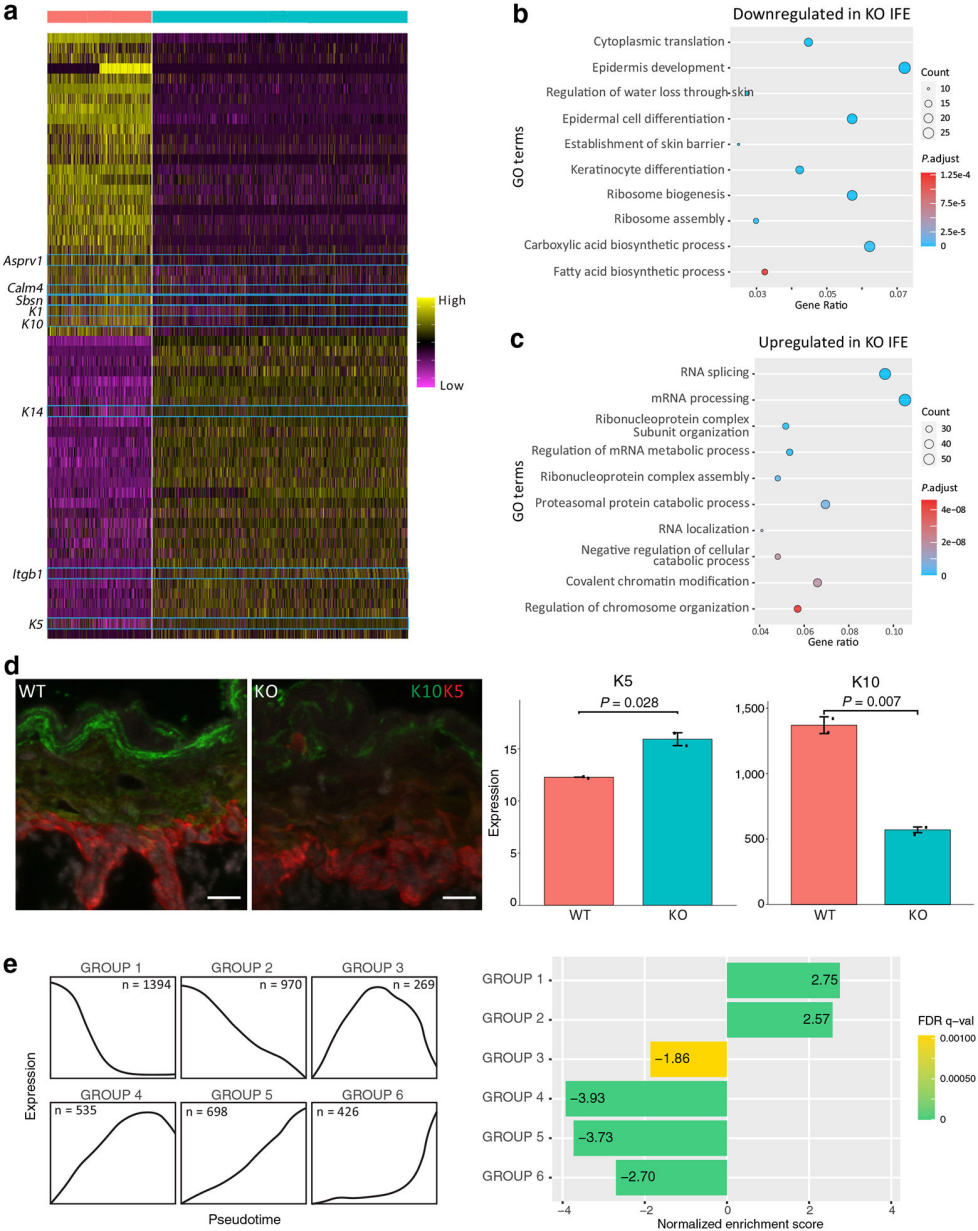
Differentiation is downregulated in the *Chrm3*^*−/−*^ IFE. (**a**) Heatmap showing 120 of the differentially expressed genes between the WT and *Chrm3*^*−/−*^ IFE. The blue boxes highlight the genes related to keratinocyte differentiation. The complete gene list is shown in Supplementary Table S3. (**b**) GO analysis reveals that keratinocyte differentiation is downregulated in the *Chrm3*^*−/−*^ IFE. (**c**) GO analysis reveals that terms related to cell proliferation are upregulated in the *Chrm3*^*−/−*^ IFE. (**d**) Distribution patterns of K5- and K10-positive keratinocytes in the epidermis of WT and *Chrm3*^*−/−*^ mice visualized by confocal microscopy of skin samples stained with anti-K5 (red) and anti-K10 (green) antibodies (bar = 20 μ m) and quantitative analysis of the intensity of fluorescence produced by each antibody within IFE showing an increase in K5 and decrease in K10 expression in KO mice. *p* - value was calculated from two-tailed *t*-test. (**e**) Expression patterns of the six groups of genes defined in Lin et al. (2020) shown on the left and enrichment score and corresponding false discovery rate *q*-value obtained from GSEA shown on the right. GO, Gene Ontology; GSEA, Gene Set Enrichment Analysis; IFE, interfollicular epidermis; K, keratin; KO, knockout; WT, wild-type.

Having established changes in epidermal differentiation and cellular composition of the *Chrm3*^*−/−*^ IFE, we next studied changes in gene expression. Differential gene expression analysis performed on all IFE populations collectively reveals downregulation of genes related to KC differentiation in the *Chrm3*^*−/−*^ IFE (Figure 3a and Supplementary Table S3). Gene Ontology (GO) analysis shows that the downregulated genes are enriched for biological processes such as epidermis development, regulation of water loss through the skin, and establishment of skin barrier (Figure 3b). These gene expression changes are not caused solely by the decrease in the proportion of differentiated KCs in the *Chrm3*^*−/−*^ IFE. GO analysis performed on downregulated genes in *Chrm3*^*−/−*^ differentiated and terminally differentiated IFE clusters alone gives similar results (Supplementary Figure S1 and Supplementary Table S4). Taken together, the decreased IFE differentiation in *Chrm3*^*−/−*^ neonatal mice is evident at both cellular and molecular levels.

By contrast, several marker genes of basal KCs such as *K14, K5*, and *Itgb1* are upregulated in the *Chrm3*^*−/−*^ IFE. The upregulated genes are overrepresented in GO biological processes such as RNA splicing, covalent chromatin modification, and regulation of chromosome organization (Figure 3c). These results point to the upregulation of cell division in the *Chrm3*^*−/−*^ IFE, which is consistent with our finding of higher proportions of S-phase and basal cells in the *Chrm3*^*−/−*^ IFE (Figure 2a and d).

The analysis of epidermal cytokeratin expression at the protein level using fluorescence staining revealed differences in the distribution of K5- and K 10-positive KCs between the epidermis of WT and *Chrm3*^*−/−*^ mice. Confocal microscopic images of the epidermis double stained for K5 and K10 revealed increased staining for K5 and decreased staining for K10 in *Chrm3*^*−/−*^mice (Figure 3d). The number of K5-positive cells within the IFE of *Chrm3*^*−/−*^ mice was significantly (n = 2–2, *P* = 0.028) higherthan that in WT mice. By contrast, the number of K10-positive cells within the IFE in *Chrm3*^*−/−*^ mice was significantly lower than in WT mice (n = 2–2, *P* = 0.007). These results support the findings of the scRNA-seq that *Chrm3* promotes epidermal differentiation.

### Basal genes are prominently upregulated in the terminally differentiated cells of the *Chrm3*^*−/−*^ epidermis

Previously, we have comprehensively characterized the P0 murine epidermal differentiation program by defining six groups of genes with distinct pseudotemporal expression patterns during IFE KC differentiation (Lin et al., 2020) (Figure 3e, left). Groups 1 and 2 genes are highly expressed in basal cells with decreasing expression as differentiation progresses. Group 3 genes are lowly expressed in basal cells, peak in the middle of differentiation, and decrease in expression toward terminal differentiation. Groups 4, 5, and 6 genes are lowly expressed in basal cells with rising expression as differentiation progresses. Using these six groups of genes as gene sets, we performed Gene Set Enrichment Analysis (Mootha et al., 2003; Subramanian et al., 2005) using genes differentially expressed in the *Chrm3*^*−/−*^ IFE as input (Supplementary Table S3). We found that group 1 and group 2 genes are overrepresented in upregulated genes and that groups 3, 4, 5, and 6 are overrepresented in downregulated genes in the *Chrm3*^*−/−*^ IFE (Figure 3e, right). This analysis indicates that *Chrm3* broadly suppresses progenitor genes and activates differentiation genes.

**Figure 4.**
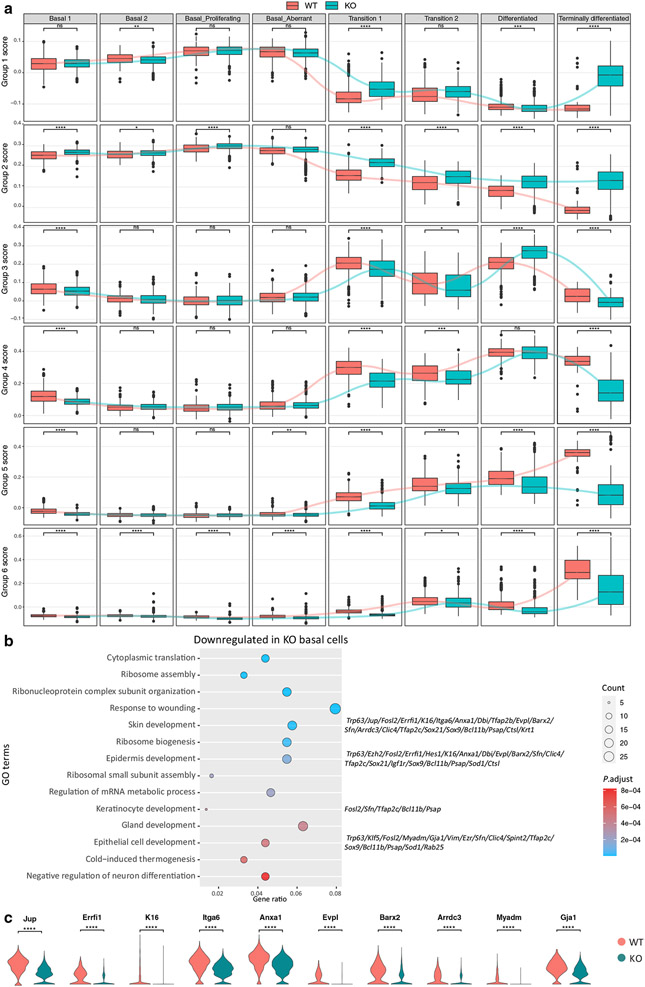
Epidermal differentiation is downregulated in suprabasal cells and adhesion molecules are downregulated in *Chrm3*^*−/−*^ basal cells. (**a**) Scoring of the cells in each differentiation stage on the basis of their expression level of six groups of genes defined in Lin et al. (2020) shows that the disturbance in the differentiation profile is most evident in the differentiated cells. *P*-value was calculated from t-test. ns: *p* > 0.05, *p ≤ 0.05, **p ≤ 0.01, *** *P* ≤ 0.001, and **** *P* ≤ 0.0001. (**b**) Gene Ontology analysis on genes downregulated in *Chrm3*^*−/−*^ basal cells show enrichment for skin development and keratinocyte differentiation. Genes contributing to the enrichment of some of the terms are listed. The complete list of differentially expressed genes in *Chrm3*^*−/−*^ versus in WT basal cells is provided in Supplementary Table S5. (**c**) Adhesion molecules are downregulated in the *Chrm3*^*−/−*^ basal cells. *P*-value was calculated from *t*-test. ns: *p* > 0.05, *p ≤ 0.05, ** *p* ≤ 0.01, ***p ≤ 0.001, and ****p ≤ 0.0001. ns, not significant; WT, wild-type.

We also used the six groups of differentiation genes to define more precisely the differentiation defect in the *Chrm3*^*−/−*^ IFE; we scored the expression of each group in each differentiation stage of the WT and *Chrm3*^*−/−*^ IFE (Figure 4a). This analysis shows that the most striking defect in the *Chrm3*^*−/−*^ IFE is in the terminally differentiated KCs where groups 1 and 2 genes are highly upregulated and groups 4, 5, and 6 genes are highly downregulated.

GRHL3 is a transcription factor that promotes differentiation (Ting et al., 2005; Yu et al., 2006) and suppresses Wnt signaling in and expansion of epidermal stem cells (Lin et al., 2020). Although the epidermal differentiation defect in *Chrm3*^*−/−*^ bears similarity to that of the *Chrm3*^*−/−*^ epidermis (Lin et al., 2020) because both mutants show accumulation of stem and progenitor cells and reduction in differentiated KCs, the marked upregulation of basal genes in the terminally differentiated KCs is unique to the *Chrm3*^*−/−*^ mutant (Supplementary Figure S2).

### Adhesion molecules are downregulated in *Chrm3*^*−/−*^ basal KCs

Because *Chrm3* is most highly expressed in the basal cell compartment (Kurzen et al., 2004; Ndoye et al., 1998), we isolated the four basal clusters and identified genes that are differentially expressed in the *Chrm3*^*−/−*^ basal cells (Supplementary Table S5). The GO terms enriched for the upregulated and downregulated genes in the *Chrm3*^*−/−*^ basal cells are similar to the ones found in differentially expressed genes and GO analysis done for the whole IFE, with processes related to cell division being upregulated (not shown) and processes related to epidermis development being downregulated (Figure 4b). A closer look at the downregulated genes contributing to GO terms such as skin development, epidermis development, KC development, and epithelial cell development reveals the downregulation of genes contributing to extracellular matrix formation, intercellular adhesion, and cell arrangement, including *Jup, Evpl, Barx2, Arrdc3*, and *Gja1* (Figure 4c). The downregulations of adhesion molecules can explain, in part, the increase in intercellular space between *Chrm3*^*−/−*^ basal cells (Figure 1a).

## DISCUSSION

In this study, we used *Chrm3*^*−/−*^ mice and scRNA-seq to characterize the role of non-neuronal ACh in epidermal gene expression and differentiation. We found that *Chrm3* promotes the expression of cell–cell adhesion molecules in the basal cell layer and differentiation throughout the epidermis. These findings support the idea that the cytotransmitter ACh—produced and released by epidermal KCs and signaling through the CHRM3 subtype expressed in epidermal stem cells—has an important epidermal developmental role. This study also sheds new light on the pathophysiology of pemphigus vulgaris where some patients develop autoantibodies inactivating CHRM3 through receptor desensitization (Chernyavsky et al., 2022). Patients with pemphigus vulgaris feature some of the same alterations in adhesion, epidermal stem cell proliferation, and differentiation as observed in the experimental model of *Chrm3* inactivation. Similar to the epidermal changes in *Chrm3*^*−/−*^ mice, anti-CHRM3 pemphigus autoantibodies upregulate K5 and downregulate K10 (Chernyavsky et al., 2022) in mice—findings that are mirrored in the epidermis of patients with pemphigus vulgaris (Williamson et al., 2006).

Changes in the cellular composition of the *Chrm3*^*−/−*^ epidermis support the prodifferentiation role of *Chrm3* in epidermal stem cells. The proportion of undifferentiated basal cells, including proliferating basal cells, increases. By contrast, the proportion of differentiated epidermal cells decreases (Figures 1e and 2a and d). Morphologic analyses support these scRNA-seq findings. The number of basal epidermal cells (Figure 1b), the epidermal thickness (Figure 1a and b), and the basal cell proliferation (Figure 2e) are increased, whereas the expression of K10 differentiation marker is decreased (Figure 3d). Our gene expression analysis showed that downregulated genes in the differentiated cells of the *Chrm3*^*−/−*^ IFE play a role in epidermal differentiation (Figure 3a and b and Supplementary Figure S1). By contrast, upregulated genes in the *Chrm3*^*−/−*^ IFE facilitate the proliferation of basal cells (Figure 3a and c and Supplementary Figure S1).

The mechanisms underlying the prodifferentiation effects of *Chrm3* remain unknown, but given that *Chrm3* is expressed to the highest levels in basal cells, we believe that it acts at the level of epidermal stem cells. Possibly, the primary function of *Chrm3* is to promote the expression of cell–cell and cell–matrix adhesion molecules. Consistent with this idea, increased intercellular spaces between basal KCs are a prominent feature of the *Chrm3*^*−/−*^ epidermis (Figure 1a). Also consistent with this finding, we noted decreased expression of a number of important cell–cell and cell–matrix adhesion molecules in the *Chrm3*^*−/−*^ basal cells (Figure 4a and b). Loss of cell–cell adhesion may impair the ability of basal KCs to undergo differentiation and promote proliferation. In other cell types, it has been shown that *Chrm3* controls the assembly of the cytoskeleton, stimulates the formation of cell–cell and cell–substrate attachments, and inhibits cell proliferation (Chernyavsky et al., 2004; Shafer et al., 1999; Shafer and Williams, 2004; Strassheim et al., 1999; Williams et al., 1993).

Many of the downregulated genes in the *Chrm3*^*−/−*^ basal cells play important roles in extracellular matrix formation, intercellular adhesion, and cell arrangement. *Jup* encodes plakoglobin, which forms adherence junctions and facilitates the formation of desmosomes. *Itga6* participates in the production of integrin, which is important for cell–substrate adhesion. *Evpl* encodes a hemidesmosome component. Autoimmunity to envoplakin is associated with paraneoplastic pemphigus (also known as paraneoplastic autoimmune multiorgan syndrome) (Nguyen et al., 2001). *Barx2* plays a critical role in cell adhesion and cytoskeleton remodeling, and its knockdown stimulates cell proliferation in human bronchial epithelial cells (Chen et al., 2018); *Arrdc3* negatively regulates integrin *β*_4_. *Gja1* encodes connexin 43, which is important for gap junctions between KCs and is targeted by pemphigus autoimmunity (Abreu-Velez et al., 2011).

We also observed a significant decrease in the proportion of Langerhans cells in the *Chrm3*^*−/−*^ epidermis. It has been previously noted that activation of the CHRM3 is essential for optimal immune responses, including both T helper 1 and T helper 2 cytokine production. *Chrm3* deficiency in mice significantly abrogates the ability to launch an effective adaptive immune response to helminth and bacterial infections (Darby et al., 2015; McLean et al., 2016).

In addition to pemphigus, altered signaling of autocrine/paracrine ACh through CHMR3 has been shown in breast, colorectal, and several other cancers (Chen et al., 2019; Lin et al., 2014; Tolaymat et al., 2019), asthma and chronic obstructive pulmonary disease (Yamada and Ichinose, 2018), immune-mediated inflammation (Kawashima and Fujii, 2004), Sjögren syndrome (Fox et al., 2000), cholinergic urticaria (Tokura, 2016), wound reepithelialization (Chernyavsky et al., 2004), and diabetes (Kruse et al., 2013) as well as gastrointestinal and urinary bladder functions (Ehlert et al., 2012; Yamanishi et al., 2001). These and some other human diseases may therefore benefit from pharmacologic modulation of CHRM3.

We previously studied the role of GRHL3 in epidermal differentiation (Kudryavtseva et al., 2003; Lin et al., 2020; Yu et al., 2006) and observed the expansion of basal cells and decreased number of differentiated cells in the *Grhl3*^*−/−*^ epidermis. Although we also observed these features in the *Chrm3*^*−/−*^ epidermis, we noticed striking differences between the differentiation defects in these two mutants. In particular, the gene expression programs associated with basal cells were uniquely upregulated in the terminally differentiated KCs of the *Chrm3*^*−/−*^ epidermis (Supplementary Figure S2). We speculate that the combined deletion of *Ghrl3* and *Chrm3* would result in an additive effect with increased expansion of basal KCs and a reduced number of differentiated cells.

In conclusion, *Chrm3* plays an important role in epidermal development, promoting adhesion of basal cells, suppressing basal cell proliferation, and promoting epidermal differentiation.

## MATERIALS AND METHODS

### Mice and reagents

The *Chrm3*^*−/−*^ mice were a generous gift from Jurgen Wess (Laboratory of Bioorganic Chemistry, Molecular Signaling Section, National Institute of Diabetes and Digestive and Kidney Diseases, National Institutes of Health, Bethesda, MD). This KO mouse line has been used in our previous experiments (e.g., Chernyavsky et al., 2004).

The Ki-67 mouse antibody was purchased from GeneTex (dilution 1:500). The K5 rabbit antibody was purchased from Abcam (Cambridge, United Kingdom) (dilution 1:500). The K10 mouse antibody was from Covance (Princeton, NJ) (dilution 1:500).

All protocols were approved by the University Laboratory Animal Resources at the University of California, Irvine (Irvine, CA).

### scRNA-seq experiments

#### Sample collection and sequencing.

Sample collection was done as previously described (Lin et al., 2020). In brief, skin samples were collected from P0 *Chrm3*^*−/−*^ mice and incubated overnight in epidermal separation buffer. The epidermis was then separated from the dermis, and the suspension of epidermal cells was passed through a 40-*μ* M filter. Dead cells were removed using Dead Cell Removal kit.

University of California, Irvine Genomic High Throughput Facility prepared the Chromium Single Cell, version 3.1 (10x Genomics, Pleasanton, CA) libraries, which were sequenced with Illumina NovaSeq6000.

### scRNA-seq data analysis

Raw sequencing files were processed using Cell Ranger 3.0.2 with the MM10 reference as stated in Lin et al. (2020).

The *Chrm3*^*−/−*^ dataset (two mice) and the previously published WT dataset (Lin et al., 2020) (two mice) were processed in R using Seurat, version 3 (Stuart et al., 2019) according to the vignette.

For all samples, only cells with 900–7,700 genes and <10% mitochondrial genes were kept for further analysis. Each of the four samples was individually log normalized, and 2,000 highly variable genes were identified before integration. Integration was performed according to the Seurat standard integration workflow, and the features.to.integrate variable was set to include all genes in the datasets. The integrated dataset was then scaled, and principal component analysis was done. Clustering analysis on the integrated dataset used the Louvain algorithm, and the output was visualized with UMAP.

For marker genes for each cell type in the skin (or differentiation stages in the IFE), we only ran the differential gene expression test on genes that are expressed in >25% of the cell-type population, and only the genes with <0.05 adjusted and >0.25 log fold change were reported.

For differential gene expression tests between the two genotypes, WT and *Chrm3*^*−/−*^, Wilcoxon rank sum tests were performed, and any genes with <0.05 adjusted *P*-values were used for GO analysis.

GO analyses were performed using ClusterProfiler in R (Yu et al., 2012). Gene Set Enrichment Analysis was performed on the desktop software Gene Set Enrichment Analysis, version 4 (Mootha et al., 2003; Subramanian et al., 2005). Gene set scores were added to scRNA-seq datasets using the AddModuleScore function provided in Seurat.

### Other methods

The number of nucleated basal cells within the IFE was measured within the 12 × 100 *μ* m rectangle frame applied to the images of IFEs taken at magnification ×20. The number of Ki-67–postive cells within the IFE was measured within the 70 × 100 *μ* m rectangle frame applied at ×20. The intensities of fluorescence produced by anti-K5 and anti-K10 antibodies were measured below the stratum corneum within IFE, the background nonspecific fluorescence was subtracted, and the resultant values were divided by the number of DAPI-positive cell numbers inside the analyzed area.

### Statistical analysis

The data were analyzed using *t*-test with a significance cutoff value of 0.05 and presented as mean ± standard error. All statistical analyses and graphs were done in R.

## Supplementary Material

Supplementary Figure S1

Supplementary Figure S2

Supplementary Tables 1-5

## Data Availability

The single-cell RNA-sequencing data of the two wild-type epidermis samples were previously published in Lin et al. (2020) and were deposited in the Gene Expression Omnibus database under GSE154579 (https://www.ncbi.nlm.nih.gov/geo/query/acc.cgi?acc=GSE154579). The single-cell RNA-sequencing data of the two *Chrm3*^*−/−*^ samples are available in the Gene Expression Omnibus database under GSE201885 (https://www.ncbi.nlm.nih.gov/geo/query/acc.cgi?acc=GSE201885).

## Abbreviations:

ACh: acetylcholine
GO: Gene Ontology
IFE: interfollicular epidermis
K: keratin
KC: keratinocyte
KO: knockout
mAChR: muscarinic acetylcholine receptor
P: postnatal day
scRNA-seq: single-cell RNA-sequencing
UMAP: Uniform Manifold Approximation and Projection
WT: wild-type
