# Supplementary material for: The M3 Muscarinic Acetylcholine Receptor Promotes Epidermal Differentiation
Source: J Invest Dermatol. Author manuscript; Available in PMC 2023 Jan 19. (PMC9851810; doi:10.1016/j.jid.2022.06.013)
Supplement: Supplementary Figure S1 [file NIHMS1854616-supplement-Supplementary_Figure_S1.pdf]

SUPPLEMENTARY MATERIALS

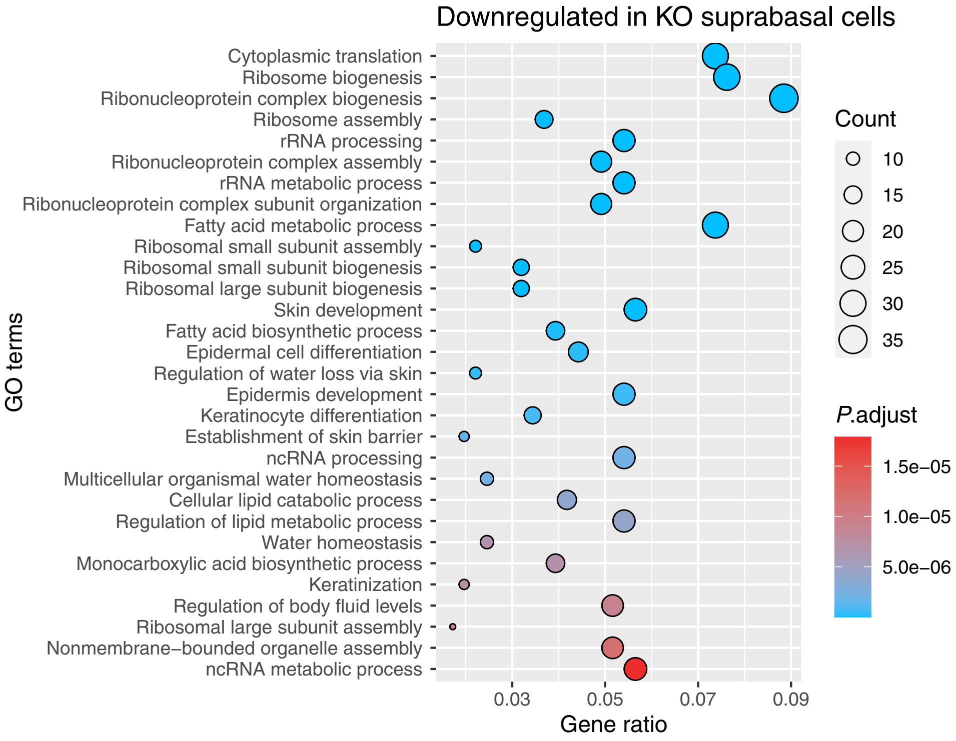

Supplementary Figure S1. Gene Ontology results obtained from genes that are downregulated in the Chrm3$^{-/-}$ differentiated and terminally differentiated cells alone show that the downregulation of differentiation we observe when comparing Chrm3$^{-/-}$ and WT IFE is not solely due to the decrease in the proportion of differentiated cells in the KO. IFE, interfollicular epidermis; KO, knockout; WT, wild-type.
