# Supplementary material for: The M3 Muscarinic Acetylcholine Receptor Promotes Epidermal Differentiation
Source: J Invest Dermatol. Author manuscript; Available in PMC 2023 Jan 19. (PMC9851810; doi:10.1016/j.jid.2022.06.013)
Supplement: Supplementary Figure S2 [file NIHMS1854616-supplement-Supplementary_Figure_S2.pdf]

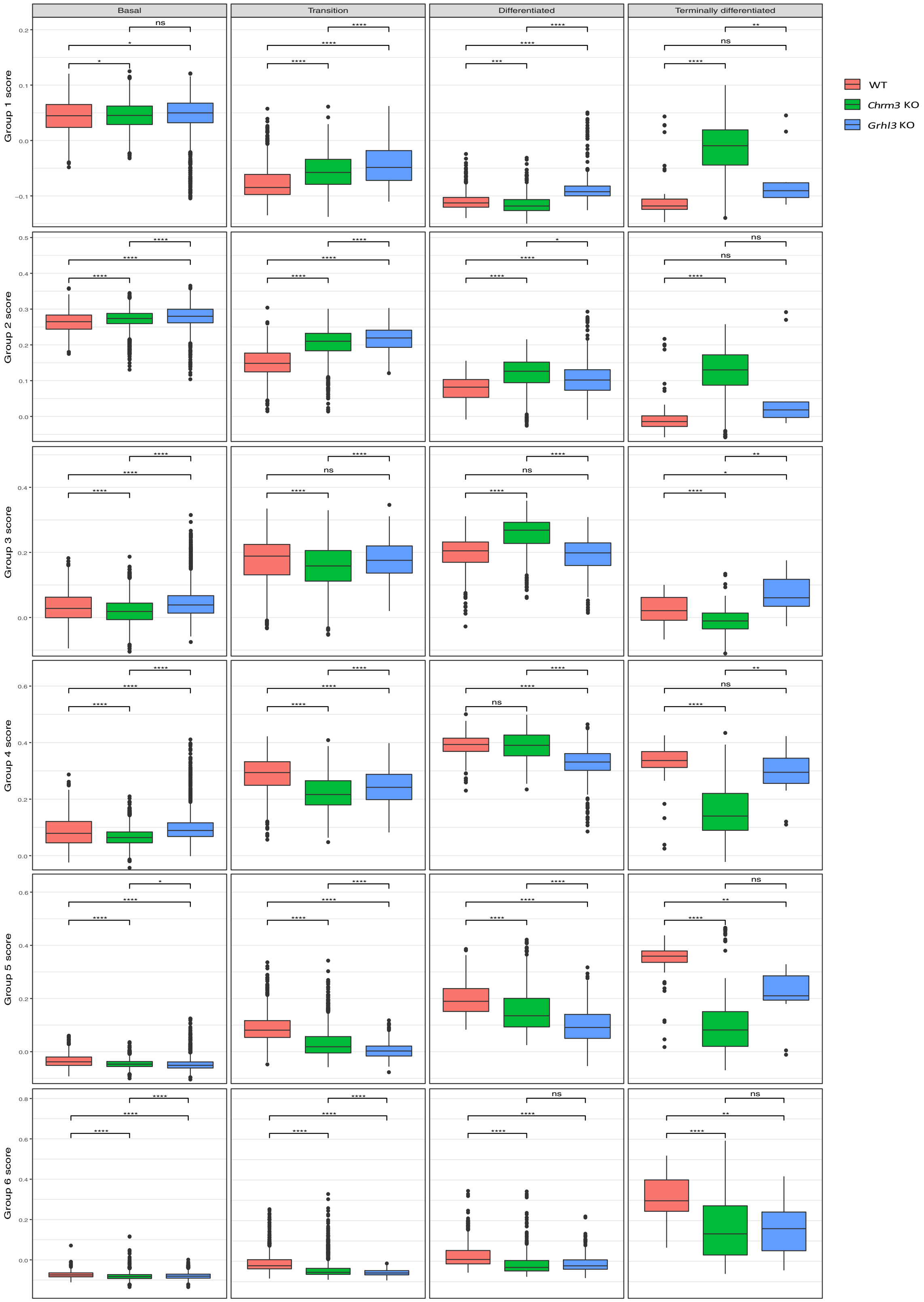

Supplementary Figure S2. Scoring of the cells in each differentiation stage on the basis of their expression level of six groups of genes defined in Lin et al. (2020) shows that Chrm3$^{-/-}$ and Grhl3$^{-/-}$ cause similar differentiation disruptions (upregulation of genes associated with basal cells and downregulation of genes associated with suprabasal cells). The defects caused by *Grhl3* $^{-/-}$ is not as prominent as the defects caused by *Chrm3* $^{-/-}$. ns: $p > 0.05$. *$p \leq 0.05$, ** $p \leq 0.01$, ***$p \leq 0.001$, and ****$p \leq 0.0001$. KO, knockout; ns, not significant.
